# West Chicago Medical Society

**Published:** 1879-05

**Authors:** W. T. Belfield

**Affiliations:** Secretary


### ARTICLE IX.

THE WEST CHICAGO MEDICAL SOCIETY met at the Washingtonian Home, Monday evening, March 24th.

Dr. D. R. Brower reported three cases of Traumatic insanity, exemplifying, as the doctor thought, three points: First, that the insanity may appear at a long interval after the cranial injury.

Second, that in the first manifestations the emotional aberrations predominate, later the intellectual. Third, that acute meningitis is not an essential feature of the disease. The doctor discussed at some length, the medico-legal phases of this condition.

Dr. Holmes, the subject being thrown open to the society, narrated a case similar in its history and bearings, to those of Dr. Brower. Dr. Lyman mentioned another; and a second one in which insanity occurred in an individual who had suffered an injury to the spinal column, the cord having apparently escaped injury. Dr. Brower mentioned incidentally a case of insanity following hernia—the insanity as well as the hernia being relieved by a truss.

Dr. Salisbury narrated a case of periodical mania, following at a considerable interval a cranial injury. The mania ceased after the free exhibition of quinine.

Dr. Bridge mentioned the case of a patient who received an injury on the head. Some time subsequently he became suddenly and violantly insane. In a short time he recovered, complaining only of pain in the head. His intellect is now somewhat impaired.

Dr. Strong presented a specimen showing anchylosis of the spine—a case of recovery from Potts' disease. The doctor referred to another specimen in his possession, which was still more remarkable.

Dr. Earle presented a specimen of intus-susception, in which there had been none of the ordinary symptoms, except vomiting. The autopsy showed no evidence of inflammatory action—not even an effusion.

### OBJECTIVE SOUNDS FROM THE EARS.

Dr. Holmes presented the abstract of a case, in which a young lady 17 years of age, pale, weak, often troubled with a cough, had suffered since early childhood from a peculiar, involuntary spasmodic contraction of the pharyngeal muscles. These spasms, occurring about forty times a minute, resembled the act of swallowing. Synchronous with each spasm was heard very distinctly, eighteen inches from the patient's left ear, a peculiar clicking sound—much less distinctly from the right ear. Listening to the patient's mouth, wide open, revealed the same sound, but faint and as if coming from a distance.

Dr. E. F. Ingals made repeated and careful laryngoscopic examinations of the fauces, and observed a separation of the lips of the Eustachian tubes with each muscular spasm, and with each sound.

The throat presented a normal appearance, the region of the mouths of the Eustachian tubes, however, being bathed with a slight muco-purulent secretion.

The external meatus was normal. The membranæ tympani were each thick and opaque at their upper portions, and thin and translucent at their middle and lower segments. With each spasm was a slight motion of the left membrane. The use of quinine and iron, cod liver oil and Fowler's solution internally, exercise and freedom from her previous close confinement, with saline solutions for the nostrils, after some months, improved the general health, but effected no change in the local symptoms.

The reporter was inclined to believe the sounds were chiefly caused by spasms of the tensor tympani muscle. The separation of the moist lips of the Eustachian tubes might increase the intensity. Such cases are very rare ; a few are mentioned in special journals of otology. In the *Boston Medical and Surgical Journal*, November 3, 1877, is reported by Prof. Henry J. Bigelow, a case in some respects analogous. The sounds seemed, however, to proceed from the throat. Dr. Holmes stated that he once had the opportunity of examining this case. He had been informed by Prof. Bigelow, that this patient, as also another similarly affected, finally recovered.

Dr. Chris. Fenger, pathologist to the county hospital, exhibited some morbid specimens obtained from a recent patient. When he opened the body, the doctor had no history of the case, except that the patient had presented typhoid symptoms. Opening the abdomen, he found infarctions in the subserous tissue of the intestines, in the spleen and kidneys. The source of these emboli was found in an ulcerous endocarditis, both sets of semilunar valves presenting an abundant deposit. Upon examining further, an embolus was found in the left middle meningeal artery, which had caused an extensive infarct ; and several small infarcts were found in each retina (illustrating the importance of the ophthalmoscope in the diagnosis of heart disease). The doctor thought, from the fact that some of the infarcts were breaking down into abcesses, that the cause of the endocarditis was septicæmia. There was no evidence of acute rheumatism, nor of any surgical operation. Careful search, however, revealed a suppurating synovitis at the bottom of a large bunion. Dr. Fenger said that this might have been the starting point of the whole disease. As proof that this was a blood disease, the doctor exhibited under the microscope some of the exudate from the heart valves, in which were myriads of micro-cocci.

W. T. Belfield, *Secretary.*

